# A new suite of reporter vectors and a novel landing site survey system to study *cis*-regulatory elements in diverse insect species

**DOI:** 10.1038/s41598-024-60432-9

**Published:** 2024-05-02

**Authors:** Kevin D. Deem, Marc S. Halfon, Yoshinori Tomoyasu

**Affiliations:** 1 Department of Biology, Miami University, Oxford, OH 45056 USA; 2 Department of Biochemistry, University at Buffalo-State University of New York, Buffalo, NY 14203 USA; 3 Present Address: Department of Biology, University of Rochester, Rochester, NY 14627 USA

**Keywords:** Gene regulatory network, *Cis*-regulatory elements, Enhancers, Reporter assay, *Drosophila melanogaster*, *Tribolium castaneum*, Development, Gene regulation

## Abstract

Comparative analyses between traditional model organisms, such as the fruit fly *Drosophila melanogaster*, and more recent model organisms, such as the red flour beetle *Tribolium castaneum*, have provided a wealth of insight into conserved and diverged aspects of gene regulation. While the study of *trans-*regulatory components is relatively straightforward, the study of *cis-*regulatory elements (CREs, or enhancers) remains challenging outside of *Drosophila*. A central component of this challenge has been finding a core promoter suitable for enhancer-reporter assays in diverse insect species. Previously, we demonstrated that a *Drosophila* Synthetic Core Promoter (DSCP) functions in a cross-species manner in *Drosophila* and *Tribolium*. Given the over 300 million years of divergence between the Diptera and Coleoptera, we reasoned that DSCP-based reporter constructs will be useful when studying *cis*-regulation in a variety of insect models across the holometabola and possibly beyond. To this end, we sought to create a suite of new DSCP-based reporter vectors, leveraging dual compatibility with *piggyBac* and PhiC31-integration, the 3xP3 universal eye marker, GATEWAY cloning, different colors of reporters and markers, as well as Gal4-UAS binary expression. While all constructs functioned properly with a *Tc*-*nub* enhancer in *Drosophila*, complications arose with tissue-specific Gal4-UAS binary expression in *Tribolium*. Nevertheless, the functionality of these constructs across multiple holometabolous orders suggests a high potential compatibility with a variety of other insects. In addition, we present the piggyLANDR (*piggyBac*-LoxP AttP Neutralizable Destination Reporter) platform for the establishment of proper PhiC31 landing sites free from position effects. As a proof-of-principle, we demonstrated the workflow for piggyLANDR in *Drosophila*. The potential utility of these tools ranges from molecular biology research to pest and disease-vector management, and will help advance the study of gene regulation beyond traditional insect models.

## Introduction

A primary goal of biology is to piece together how genes regulate each other, and how these regulatory interactions control biological processes^1,2^. Gene regulation can be broadly decomposed into two categories: genes are regulated in *trans* via DNA-binding proteins, such as transcription factors, and in *cis,* via DNA sequences present at the gene locus^3^. Enhancers comprise a subclass of these *cis*-regulatory elements, and integrate information from available *trans* factors to determine gene expression patterns^3^.

The collection of genes and their regulatory interactions involved in a given biological process is called a gene regulatory network (GRN). Research centered on the role of GRNs in the fields of development, evolution, and disease has started to offer a more comprehensive understanding of how biological processes are controlled through intricately entwined actions of *cis* and *trans* factors^1,2,4,5^. The development of molecular tools that allow the dissection of genetic regulation in *cis* and *trans* in tractable model systems is, of course, a prerequisite for investigations of this nature^1,2,4,6,7^.

*Drosophila melanogaster* is currently by far the best-suited model insect for the analysis of both *cis* and *trans* properties of GRNs^6,7^. However, to obtain a more comprehensive understanding of how GRNs operate and evolve, it will be critical to go beyond *Drosophila* and study a wide variety of insects and other species. Dissecting the *trans* properties of GRNs is relatively straightforward even in species outside of *Drosophila*. This is largely due to the relatively easy application of RNA interference (RNAi)-based gene knockdown^8,9^, and more recently Clustered Regularly Interspaced Short Palindromic Repeats (CRISPR)/ CRISPR-associated protein 9 (Cas9) -based gene knockout^10–12^, in many insects. However, the study of *cis* properties presents a considerable challenge outside of *Drosophila*^7^. One major barrier to functionally dissecting *cis*-regulation in non-traditional insect models is the lack of a reliable enhancer reporter assay system^6,7^.

In an enhancer reporter assay, a putative enhancer is placed upstream of a core promoter and a reporter gene, such as Enhanced Green Fluorescent Protein (EGFP). This construct can then be integrated into the genome to visualize the activity of the enhancer through expression of the reporter^6^. One of the crucial components that have restricted the use of this approach primarily to *Drosophila* is a reliable core promoter. The *Drosophila melanogaster-hsp70* (*Dm-hsp70*) core promoter has historically been the most commonly used core promoter in insect reporter vectors^6,7^. While this core promoter can function with some enhancers in non-*Drosophila* species, such as the 3xP3 artificial eye enhancer or those responsible for enhancer traps^13,14^, it does not work reliably with others, such as the yeast Upstream Activation Sequence (UAS) in the red flour beetle *Tribolium castaneum*^15–17^ (also see ref^7^ for details about previously used core promoters in insect species).

Previously, we demonstrated that a different core promoter, a variant of the *Drosophila* Synthetic Core Promoter (DSCP)^18^, works well in a cross-species setting, and is capable of reporting *Drosophila* and *Tribolium* enhancer activity in transgenic *Drosophila*, as well as in transgenic *Tribolium*^19^. More recently, our DSCP-based reporter was shown to also function in transgenic butterflies (*Bicyclus anynana*) and transgenic mosquitos (*Aedes aegypti*)^20,21^. Given that there is over 300 million years of divergence time between the Coleoptera and Lepidoptera from the Diptera^22^, we reasoned that this cross-species compatibility makes the DSCP a promising candidate for reporting enhancer activity in a wide variety of insect species throughout the holometabola and possibly beyond. To this end, we have created and validated a suite of new reporter vectors utilizing the DSCP with different colors of fluorescent reporters and markers, as well as with the Gal4-UAS binary expression system, for use in non-traditional model insects. Furthermore, we made these constructs compatible with both *piggyBac*-based transgenesis^23,24^ and PhiC31site-directed integration^25^, allowing researchers to choose their transgenic method based on the availability of genetic tools in each insect species.

In addition to the choice of core promoter, the avoidance of position effects is also crucial to the reliability of enhancer-reporter assays. Position effects can arise from the activating or silencing influence of native *cis*-regulatory elements or the local chromatin state surrounding a transgenic insertion^6^. In *Drosophila*, position effects can be largely avoided thanks to an abundance of well-studied, ‘safe’ landing sites for transgenic constructs which can be utilized via PhiC31 site-directed mutagenesis^6,25,26^. Availability of such safe landing sites in non-traditional model insects would be highly beneficial for many transgenic techniques including enhancer-reporter assays. For this purpose, we present the piggyLANDR (*piggyBac* LoxP AttP Neutralizable Destination Reporter) concept, a platform for the establishment of PhiC31 landing sites free from position effects such as enhancer traps and heterochromatic silencing. piggyLANDR randomly inserts a 3xP3-ECFP marked PhiC31 attP site along with a removable Gal4-UAS-tdTomato cassette to probe potential landing sites for position effects. Activating position effects at a locus will cause Gal4-UAS-tdTomato expression from a piggyLANDR insertion through enhancer trapping, while silencing position effects will prevent UAS-tdTomato expression when a piggyLANDR line is crossed with a separate Gal4 driver. After excluding the sites with either activating or silencing position effects, ideal landing sites with no detectable position effects can have the Gal4-UAS cassette removed via Cre-loxP recombination, to be used as PhiC31 destinations for transgenic constructs. As a proof-of-principle, we demonstrate the ability of piggyLANDR to perform these functions in *Drosophila*.

The tools developed here expand the ability of researchers to explore the conserved and derived genetic regulatory interactions underlying important biological processes in diverse insect species. Furthermore, the tissue-specific control of transgene expression in non-*Drosophila* insects provides potentially valuable resources for pest management and disease vector control.

## Results

### Expanding the functionality of enhancer-reporter vectors

**Figure 1:**
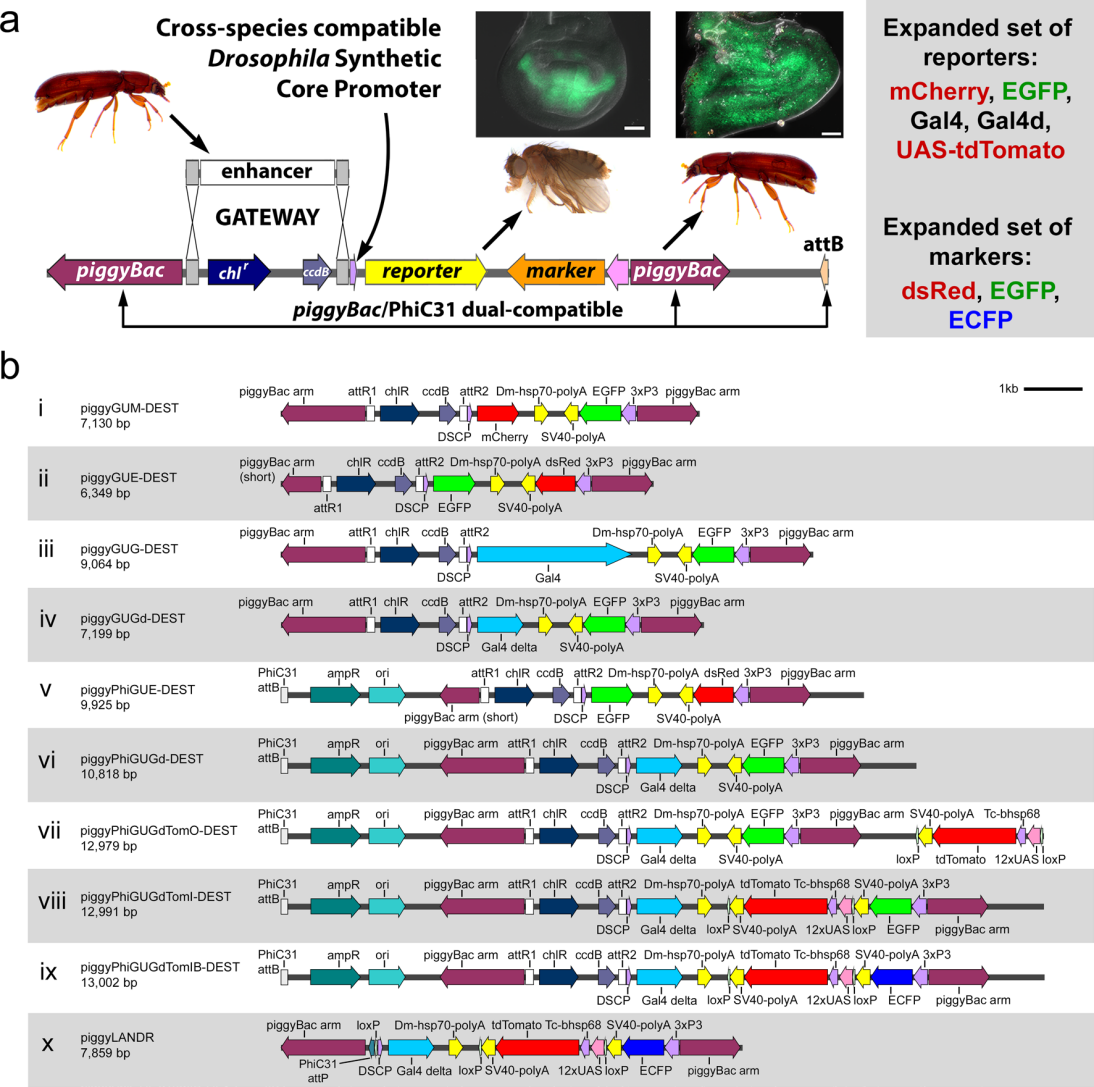
New suite of reporter vectors for diverse insects. (**a**) Expanding the functionality of the DSCP-based reporter construct by selecting different modes of transgenesis, reporter genes, and transgenic markers. A wing enhancer of *Tribolium* is shown as an example. A putative wing enhancer can be isolated from the *Tribolium* genome, cloned into one of our reporter vectors, and transformed into both *Tribolium* (i.e. the native species) via *piggyBac* transgenesis and *Drosophila* (i.e. a cross-species setting) via PhiC31 transgenesis, and evaluated for its enhancer activity using DSCP as a universal basic promoter. (**b**) Linear maps of the previously published vector piggyGUM (i) and the new constructs presented in this study (ii–x). An enhancer of interest can be cloned into the GATEWAY cassettes (attR1-attR2; i–ix) through an LR clonase reaction. Only the components relevant to reporter constructs are shown. *ampR* Ampicillin-resistance gene, *chlR* Chloramphenicol resistance gene, *ori* Replication origin.

Given the successful outcome of our DSCP-based reporter constructs across three orders of holometabolous insects^19–21^, we reasoned that expanding the functionality of the DSCP-based reporter construct will be useful when studying *cis*-regulation in diverse insect species (Fig. 1a). piggyGUM (Fig. 1b, i) is the original DSCP-based reporter construct^19^. Several aspects were modified in this study, including the mode of transgenesis, reporter genes, and transgenic markers (Fig. 1a, b ii–ix). For the mode of transgenesis, we added an attB site to our constructs, which makes them compatible with both *piggyBac*-based transgenesis^23,24^ and PhiC31 site-directed integration^25^. For reporters, we made versions with several different colors of fluorescent reporters, as well as with the Gal4-UAS binary expression system. We also changed the color of the transgenic marker, so it does not overlap with the color of the reporter. In addition, we kept the GATEWAY cloning compatibility of the original construct, allowing quick cloning of an enhancer of interest into the reporter construct.

We named these new reporter constructs based on their core components. For instance, “**piggy**” represents the presence of the *piggyBac* transposon arms for random integration^23,24^, while “**Phi**” indicates the inclusion of the PhiC31 attB site for site-directed integration^25^. Other components include a GATEWAY cassette (**G**) for easy enhancer cloning, the Universal (**U**) DSCP for reporter gene expression^18^, and the 3xP3 artificial eye promoter for the transgenic marker (not indicated in the name)^13,27^. The letter which comes after ‘U’ in the name indicates the reporter gene: **M** for mCherry, **E** for EGFP, **G** for full-length Gal4, and **Gd** for the deletion variant Gal4-delta (Table S1). Gal4-delta is a truncated version of Gal4 in which the N-terminal DNA-binding domain (DBD) and C-terminal activation domain (AD) are fused, and is required for UAS activation in *Tribolium*^16,28^. Several Gal4-delta vectors also contain a 12xUAS-tdTomato cassette flanked by loxP sites^29^ inserted either Inside (Tom**I**) or Outside (Tom**O**) of the *piggyBac* arms. The core promoter from the *Tribolium* ortholog of the *Dm-hsp70* gene (*Tc-hsp68*) is utilized in the 12xUAS-tdTomato cassette, as its functionality has been demonstrated in *Tribolium*^16^. Finally, the **B** at the end of piggyPhiGUGdTomIB indicates that the 3xP3-EGFP marker has been swapped for 3xP3-ECFP, short for the Blue color of the Enhanced Cyan Fluorescent Protein.

All enhancer-reporter vectors function as a GATEWAY destination vector, meaning that they have an [*attR1-ccdB-chl*^*r*^*-attR2*] cassette at the cloning site before GATEWAY cloning. These vectors, prior to inserting the enhancer, are designated as “DEST” (as in the “destination vector” of the LR clonase reaction). All vectors are resistant to ampicillin and chloramphenicol (*amp*^*r*^, *chl*^*r*^) and contain the *ccdB* gene (*ccdB* +) prior to GATEWAY cloning, but retain only the amp resistance after GATEWAY cloning. After an enhancer is inserted via GATEWAY cloning, the name of the enhancer is indicated after the name of the vector. For example, piggyGUE-TcNub1L is the piggyGUE vector into which the TcNub1L enhancer, a *Tribolium nubbin* (*nub*) wing enhancer (Lai et al., 2018), is inserted.

### Enhancer-reporter vector functionality in *Drosophila*

**Figure 2:**
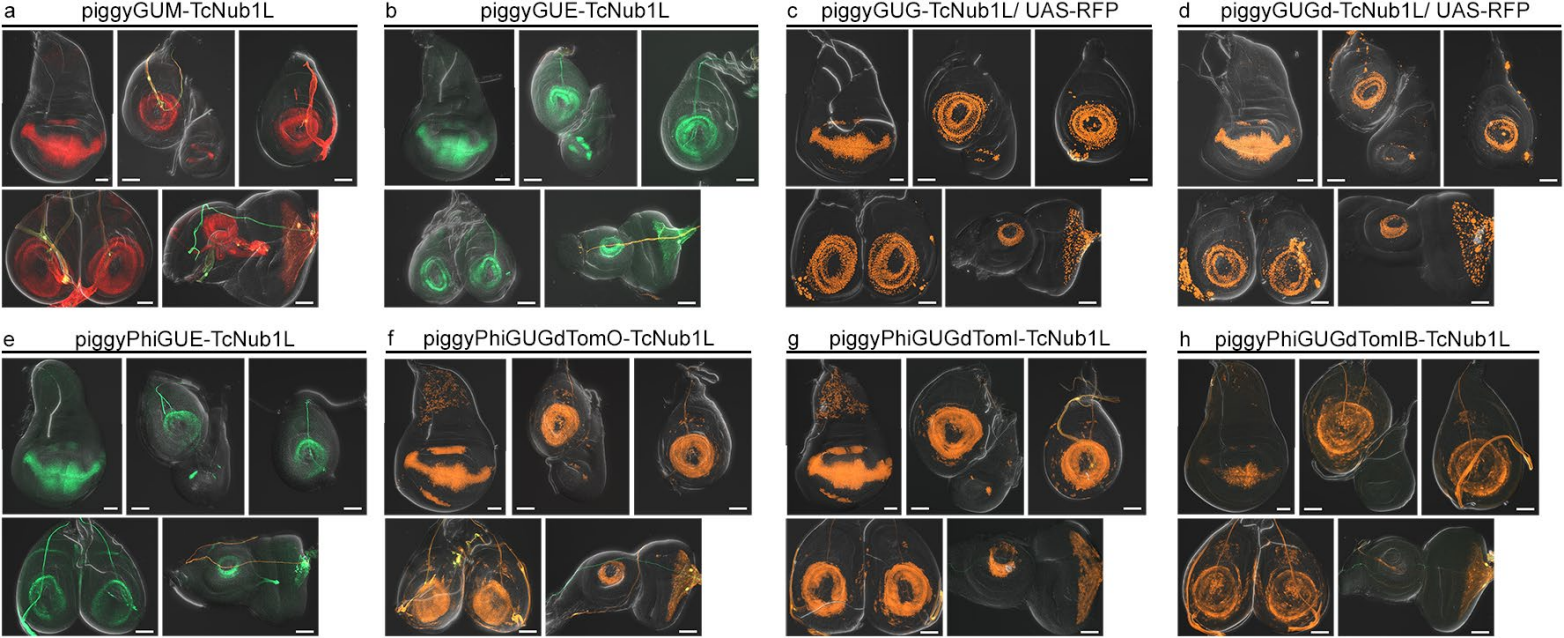
Comparison of enhancer-reporter vectors in *Drosophila* with piggyGUM-TcNub1L. (**a**–**h**) Expression driven in the *Drosophila* imaginal discs by the previously published vector piggyGUM-TcNub1L (**a**) and the new suite of reporter vectors (**b–h**). The enhancer activity was visualized by mCherry (**a**), EGFP (**b**, **e**), UAS-Red Fluorescent Protein (RFP) (**c**–**d**), and UAS-tdTomato (**f**–**h**). All new constructs (**b**–**h**) recapitulated the expression pattern driven by the previously published piggyGUM-TcNub1L (**a**) in *Drosophila* imaginal discs. Scale bars: 50 $\mu$m.

All vectors (except piggyPhiGUGd, see below) were first tested in *Drosophila* with the *Tribolium nubbin* wing enhancer (TcNub1L), via *piggyBac* random insertion when necessary, or PhiC31 integration into attP2 on chromosome 3L^25^ when possible. Two independent lines for each construct were analyzed. Those new constructs (Fig. 1b, i–v, vii–ix) faithfully recapitulated the expression pattern driven by the previously published piggyGUM-TcNub1L vector in *Drosophila* imaginal discs (Lai et al., 2018; Fig. 2). piggyPhiGUGd (Fig. 1b vi) was tested using a novel enhancer from the *Drosophila vestigial* gene (Tomoyasu Y, *unpublished data*) and recapitulated expression driven by this enhancer in the DSCP-based Gal4 vector p $\Phi$ UGG^30^. Unfortunately, piggyPhiGUGdTomIB-TcNub1L drove very weak expression (Fig. 2h), thus this vector was utilized for an alternative purpose (see the piggyLANDR section below).

### Enhancer-reporter vector functionality in *Tribolium*

**Figure 3:**
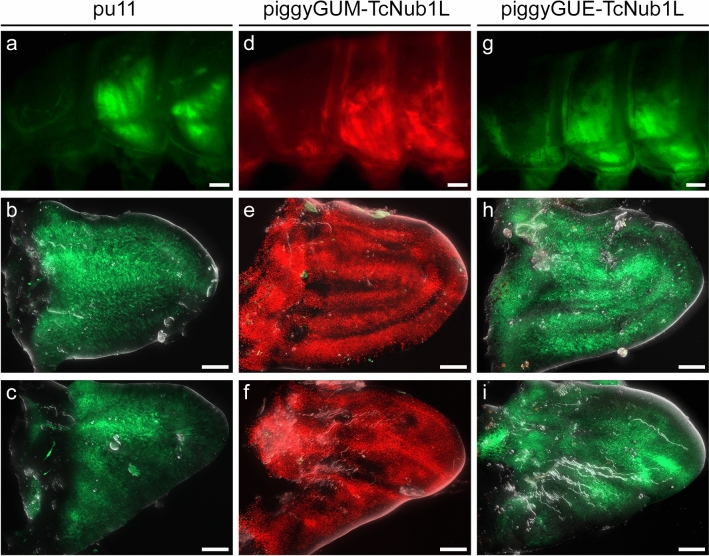
Comparison of enhancer-reporter vectors in *Tribolium* . (**a–i**) Expression of the *Tc-nub* enhancer trap line *pu11* (**a**–**c**), piggyGUM-TcNub1L (**d**–**f**), and piggyGUE-TcNub1L (**g**–**i**) in *Tribolium*. Wing expression is shown in the larval thorax (**a**, **d**, **g**), dissected elytral (**b**, **e**, **h**) and hindwing discs (**c**, **f**, **i**). The new piggyGUE construct (**g–i**) recapitulated the expression pattern of previously published piggyGUM-TcNub1L (**d**–**f**). Scale bars: 100 $\mu$m (**a**, **d**, **g**); 50 $\mu$m (**b**–**c**, **e–f**, **h–i**).

**Figure 4:**
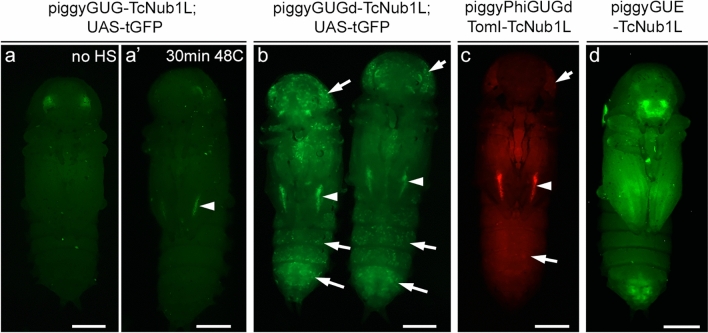
Expression of Gal4-UAS vectors in *Tribolium.* (**a, a’**) piggyGUG-TcNub1L/UAS-tGFP without heat-shock (at 30 °C, a), and with heat-shock (at 48 °C, a’). tGFP expression is seen in the wing stripe only after the heat-shock (arrowhead in a’). (**b**) piggyGUGd-TcNub1L/UAS-tGFP at 30 °C. tGFP expression is seen in the wing stripe (arrowheads), as well as in the cells scattered throughout the larval and pupal body (arrows). (**c**) piggyPhiGUGdTomI-TcNub1L at 30 °C. tdTomato expression is seen in the wing stripe (arrowhead), and also in internal tissues throughout the body (arrows). (**d**) piggyGUE-TcNub1L. The whole wing activity of TcNub1L in piggyGUE (**d**) differs significantly from its activity tested with the Gal4-UAS reporter constructs (**a–c**). Scale bars: 0.5 mm.

Select vectors were then tested in *Tribolium*, to assess the function of key novel components that are implemented in piggyGUE, piggyGUG, piggyGUGd, and piggyPhiGUGdTomI (Fig. 3, 4). piggyGUE-TcNub1L drove EGFP expression throughout the wing tissue (Fig. 3g–i), recapitulating mCherry expression driven by piggyGUM-TcNub1L (Fig. 3d–f), and overlapping the expression driven by the EYFP *Tc-nub* enhancer trap line *pu11* (Fig. 3a–c). For vectors utilizing the Gal4-UAS system, complications arose when tested in *Tribolium* (Fig. 4). It was previously reported that the full-length Gal4 protein was unable to activate a UAS-tGFP transgene in *Tribolium*, and that the deletion variant Gal4-delta is required^16^. Consistent with these findings, piggyGUG-TcNub1L failed to drive expression of a UAS-tGFP transgene at the standard rearing temperature of 30 °C (Fig. 4a), despite RT-qPCR verification that Gal4 is transcribed (data not shown). However, following a 30 min heat-shock at 48 °C, a faint stripe of tGFP expression was visible in the early pupal wing tissue (Fig. 4a’). piggyGUGd-TcNub1L strongly activated UAS-tGFP in this stripe, as well as in internal tissues that are present throughout the larval and pupal bodies (Fig. 4b). We originally hypothesized that this may be due to a position effect on the UAS-tGFP transgene. However, including a UAS-tdTomato cassette in the vector, with piggyPhiGUGdTomI-TcNub1L, yielded similar results in 5 independent lines (Fig. 4c). Wing expression remained limited to a small stripe in early pupae, and expression was still driven in internal tissues throughout the bodies, albeit at lower levels in some lines (Fig. 4c).

The expression driven by the TcNub1L enhancer in our Gal4-UAS vectors (in a small stripe in the pupal wing and ubiquitously in the internal tissues; Fig. 4), lies in stark contrast to the highly similar expression patterns driven by piggyGUM-TcNub1L and piggyGUE-TcNub1L (specific to the larval and pupal wing tissue; Fig. 3d–i). It does not appear that this discrepancy can be attributed to position effects, as it is independent of the insertion site of Gal4 and the UAS (Fig. 4). While general misbehavior of the Gal4-UAS system in *Tribolium* may be to blame, this conundrum also calls into question the true native activity of the TcNub1L enhancer (*i.e.* which reporter expression pattern should we believe as the active domain of the tested enhancer, either the Gal4-UAS expression or the direct reporter expression?). Speculative causes of this discrepancy are offered in the Discussion.

### piggyLANDR (*piggyBac* LoxP AttP neutralizable destination reporter): conceptual model

The primary goal of an enhancer-reporter assay is to reliably reproduce the activity of an enhancer at its native locus, while inserted elsewhere in the genome. A major barrier to this goal is the potential activation or silencing of the inserted transgene by *cis*-regulatory elements present in the flanking genomic sequence or by chromatin status (i.e. position effects)^6^. Additionally, transgenes may insert within exons, promoters, or other *cis*-regulatory elements, disrupting endogenous gene expression, and creating unhealthy or homozygous lethal lines^6^. Such scenarios are unfortunately unavoidable when using random transposon insertion methods, such as *piggyBac*. Alternatively, site-specific integration (such as with PhiC31) ensures that the transgene inserts only at a designated landing site^6^. In the PhiC31 system, recombination between an attP landing site in the genome and an attB site in the vector, mediated by the PhiC31 recombinase, facilitates site-specific integration of the vector into the genome^25^. Extensive work has been done in *Drosophila* to insert a multitude of attP landing sites, and then characterize position effects on each insertion^25,26^. This ability to choose a ‘safe’ landing site proved highly useful during genome-wide enhancer screens. For example, The FlyLight Project utilized the attP2 landing site, shown to be inserted in intergenic sequence relatively free from position effects^18,25,26^. Although currently unavailable outside of *Drosophila*, an abundance of safe attP landing sites, free from position effects, would greatly benefit transgenic research in other species.

**Figure 5:**
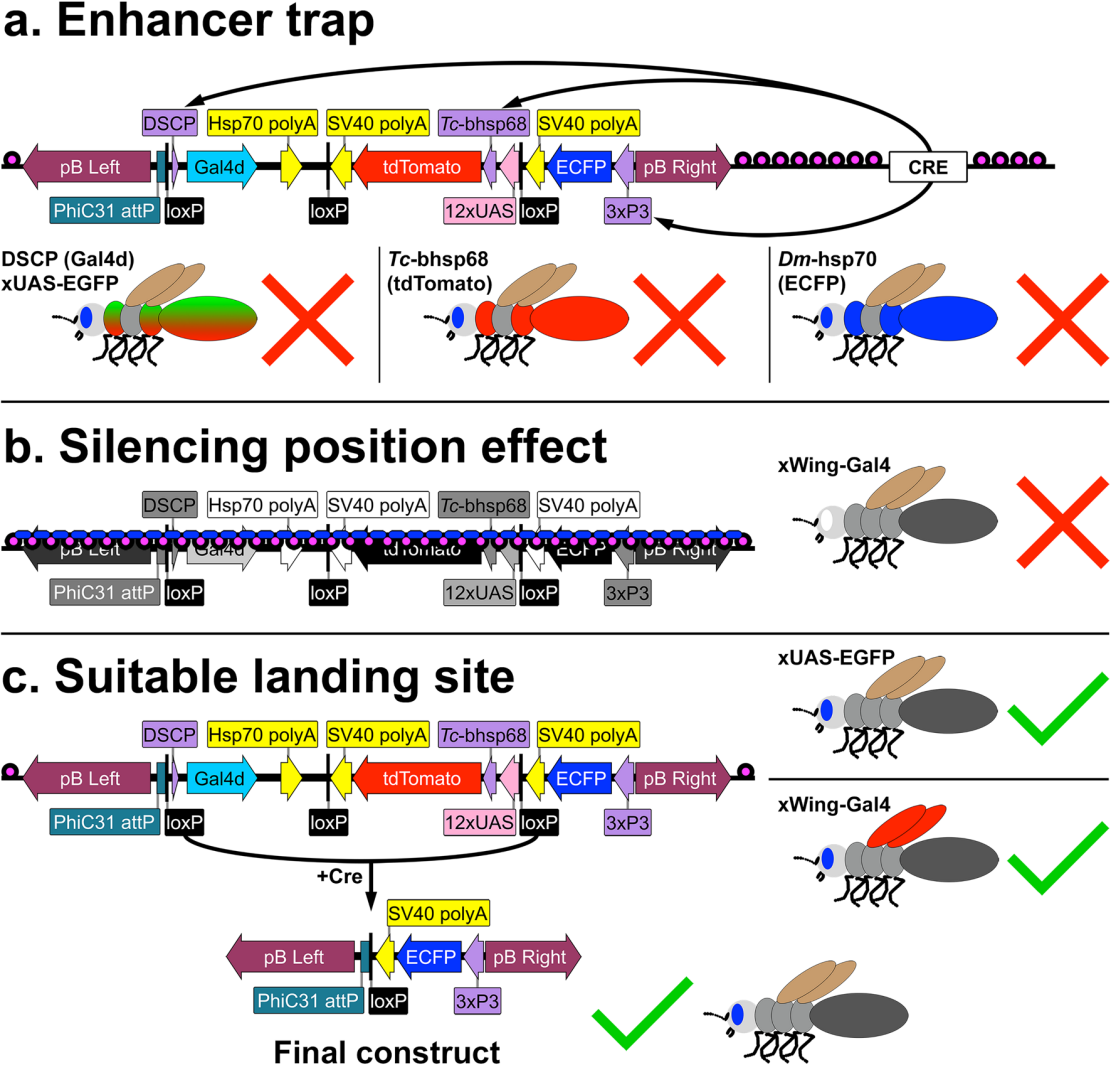
Workflow to identify position effect-free PhiC31 attP landing sites with piggyLANDR. (**a**) Enhancer traps can be detected on any of three promoters based on the expression of tdTomato, ECFP, or activation of a separate UAS-reporter by Gal4d. (**b**) Silencing position effects (including heterochromatin formation shown in the diagram) can be assessed via activation of UAS-tdTomato with a separate Gal4 driver (such as a wing-Gal4 in the diagram). (**c**) Once the ideal landing site unaffected by both enhancer trapping and silencing position effects is identified, the reporter function of the piggyLANDR construct will be removed via Cre-loxP recombination, leaving a 3xP3-ECFP marked PhiC31 attP landing site free of position effects.

We have developed a system to insert and immediately evaluate attP landing sites for position effects, which we call piggyLANDR (*piggyBac* LoxP AttP Neutralizable Destination-Reporter) (Fig. 5). piggyLANDR utilizes random *piggyBac* insertion to place PhiC31 attP landing sites throughout the genome. These landing sites are accompanied by three transgenes under the control of different core promoters which allows the assessment of position effects on each insertion (Fig. 5a). The utilization of three popular core promoters allows screening for position effects which might influence a variety of enhancer-reporter vectors. The first transgene is 3xP3-Dm-hsp-ECFP, which also serves as a fluorescent eye marker. The next two transgenes, DSCP-Gal4d and UAS-Tc-bhsp68-tdTomato, work together to additionally report activity of nearby endogenous enhancers as well as chromatin status near the insertion site. After piggyLANDR is successfully integrated into the genome, unwanted activating position effects at the insertion site can be detected via expression of ECFP outside of the eye or tdTomato anywhere in the body (Fig. 5a). For lines that exhibit no activating position effects, activation of UAS-Tc-bhsp68-tdTomato by crossing to a Gal4 driver line can be used to test for silencing effects, namely that a Gal4 driver would fail to activate UAS-tdTomato if piggyLANDR is inserted in a transcriptionally silenced locus, such as a heterochromatic region (Fig. 5b). Additionally, expression of DSCP-Gal4d but silencing of UAS-tdTomato may be detected by crossing to a UAS-GFP line (Fig. 5a,c). For lines which exhibit no activating or silencing effects, the Gal4d-UAS-tdTomato portion of piggyLANDR can be removed via Cre-loxP recombination, leaving a 3xP3-ECFP marked attP landing site, suitable for enhancer reporter assays (Fig. 5c).

### piggyLANDR: proof of principle in *Drosophila*

**Figure 6:**
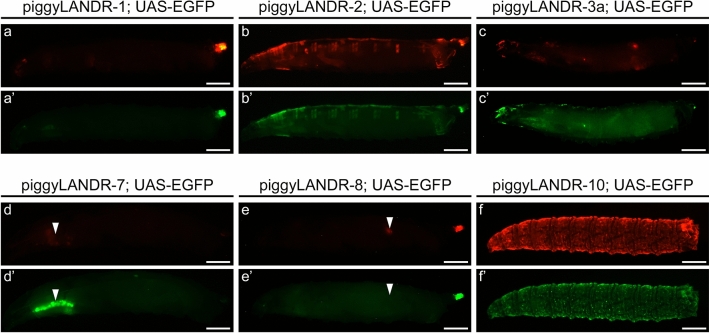
Examples of enhancer traps in piggyLANDR lines. (**a**–**f**) Enhancer trap expressions detected in the third instar larvae of six piggyLANDR lines crossed with UAS-EGFP. In most lines, UAS-tdTomato expression was highly similar to UAS-EGFP expression (i.e. enhancer trapping on DSCP-Gal4d) (**a**–**c**’, **f**, **f**’). In some lines, only EGFP (arrowheads, d, d’) (i.e. enhancer trapping on DSCP-Gal4d, with silencing on UAS-tdTomato), or tdTomato (arrowheads, e, e’) (i.e. enhancer trapping on UAS-tdTomato) was detected. The minimal overall enhancer trap expression of piggyLANDR-1 (**a**) makes this insertion site a good candidate for an ideal PhiC31 landing site free of position effects. Scale bars: 0.5 mm.

**Figure 7:**
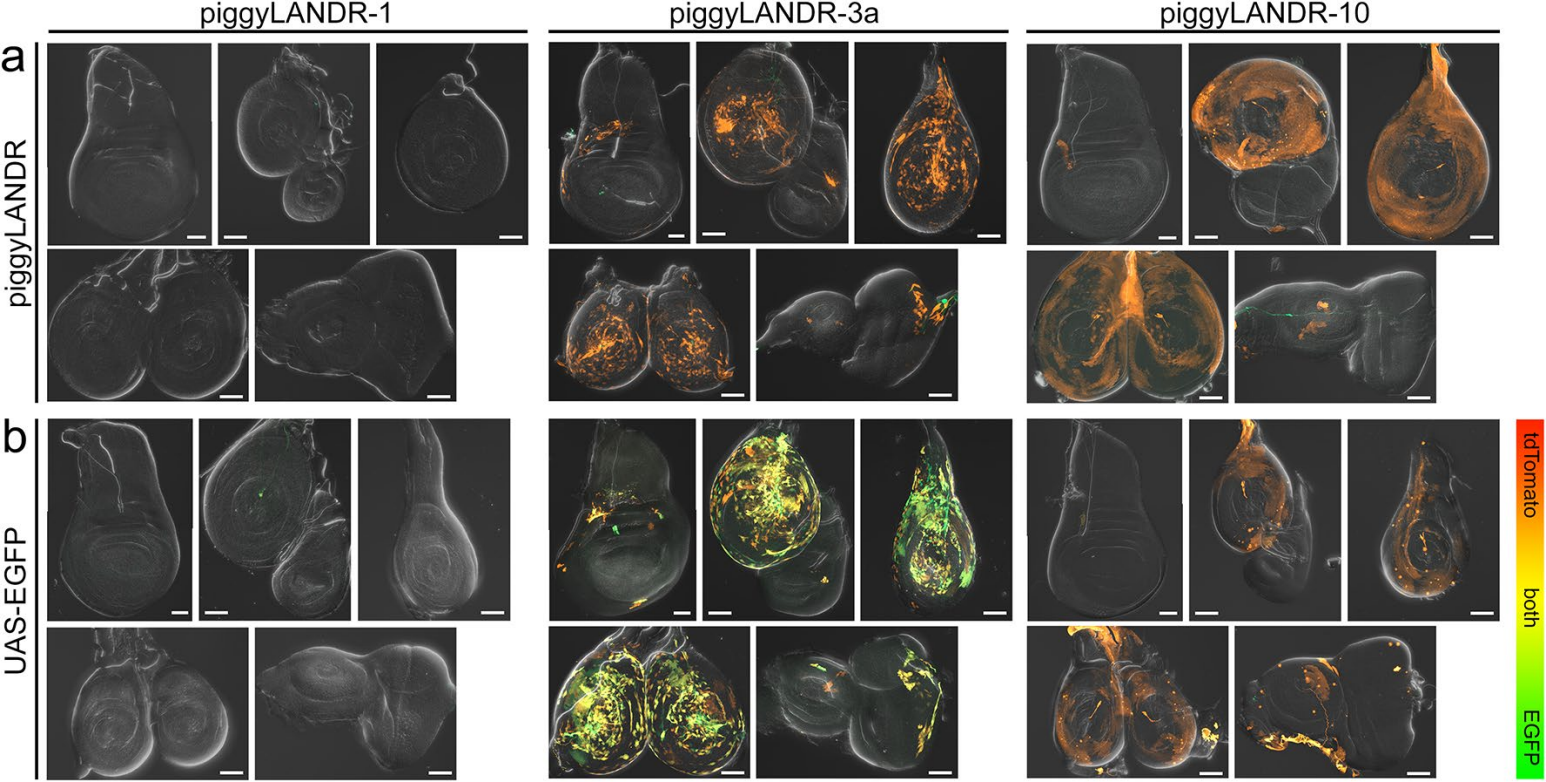
piggyLANDR enhancer traps in larval imaginal discs. (**a**) tdTomato enhancer trap expression of piggyLANDR-3a and piggyLANDR-10 in the imaginal discs. Only two out of 11 piggyLANDR lines exhibited enhancer trap expression in the imaginal discs. (**b**) EGFP expression of piggyLANDR-3a and piggyLANDR-10 when crossed to UAS-EGFP. piggyLANDR-3a had overlapping expression of EGFP and tdTomato. In contrast, piggyLANDR-10 failed to activate UAS-EGFP in the imaginal discs, suggesting an enhancer trap on UAS-tdTomato but not on DSCP-Gal4d in the imaginal discs of this line. The absence of any observable enhancer trap expression in the imaginal discs of piggyLANDR-1 makes this insertion site a good candidate for an ideal PhiC31 landing site free of position effects. Scale bars: 50 $\mu$m.

As a proof-of-principle, we performed the procedures for establishing PhiC31 landing sites with piggyLANDR in *Drosophila*. Eleven independent *piggyBac*-based insertion lines were obtained. First, we screened for any position-dependent reporter expression at the last larval stage. We excluded expression driven by the construct itself from analysis, such as the 3xP3-driven expression of ECFP in the CNS and anal pads, and the possible DSCP-driven tdTomato expression in the posterior spiracles that we observed regardless of the insertion site. Seven of the eleven lines exhibited tdTomato expression outside of these domains at the last larval stage (see Fig. 6 for examples), including two that were active in all imaginal discs analyzed (Fig. 7), indicating activating position effects at these insertion sites (Table S3).

Next, we sought to address the possibility that UAS-tdTomato expression is silenced while DSCP-Gal4d was still activated by position effects. First, all lines were crossed to UAS-EGFP to detect any Gal4d expression failing to activate UAS-tdTomato. Most lines exhibited highly similar expression patterns of UAS-tdTomato and UAS-EGFP, suggesting that there was no differential activation or silencing of DSCP-Gal4d or UAS-tdTomato (Fig. 6a–c’, f–f’, Fig. S1, Table S3 green cells). One line (piggyLANDR-7) exhibited UAS-EGFP expression in the salivary glands where there was no UAS-tdTomato expression (Fig. 6d, d’), suggesting a silencing position effect on the UAS-tdTomato, but not on DSCP-Gal4d, at this insertion site (see Table S3 yellow cells for all such cases). Conversely, two lines exhibited tdTomato expression in domains with no activation of the UAS-EGFP: piggyLANDR-8 in the larval testes (Fig. 6e, e’) and piggyLANDR-10 in the larval imaginal discs (Fig. 7; Table S3 orange cells). This suggests that an activating position effect (enhancer trap) is influencing the UAS-tdTomato but not DSCP-Gal4d in those lines.

**Figure 8:**
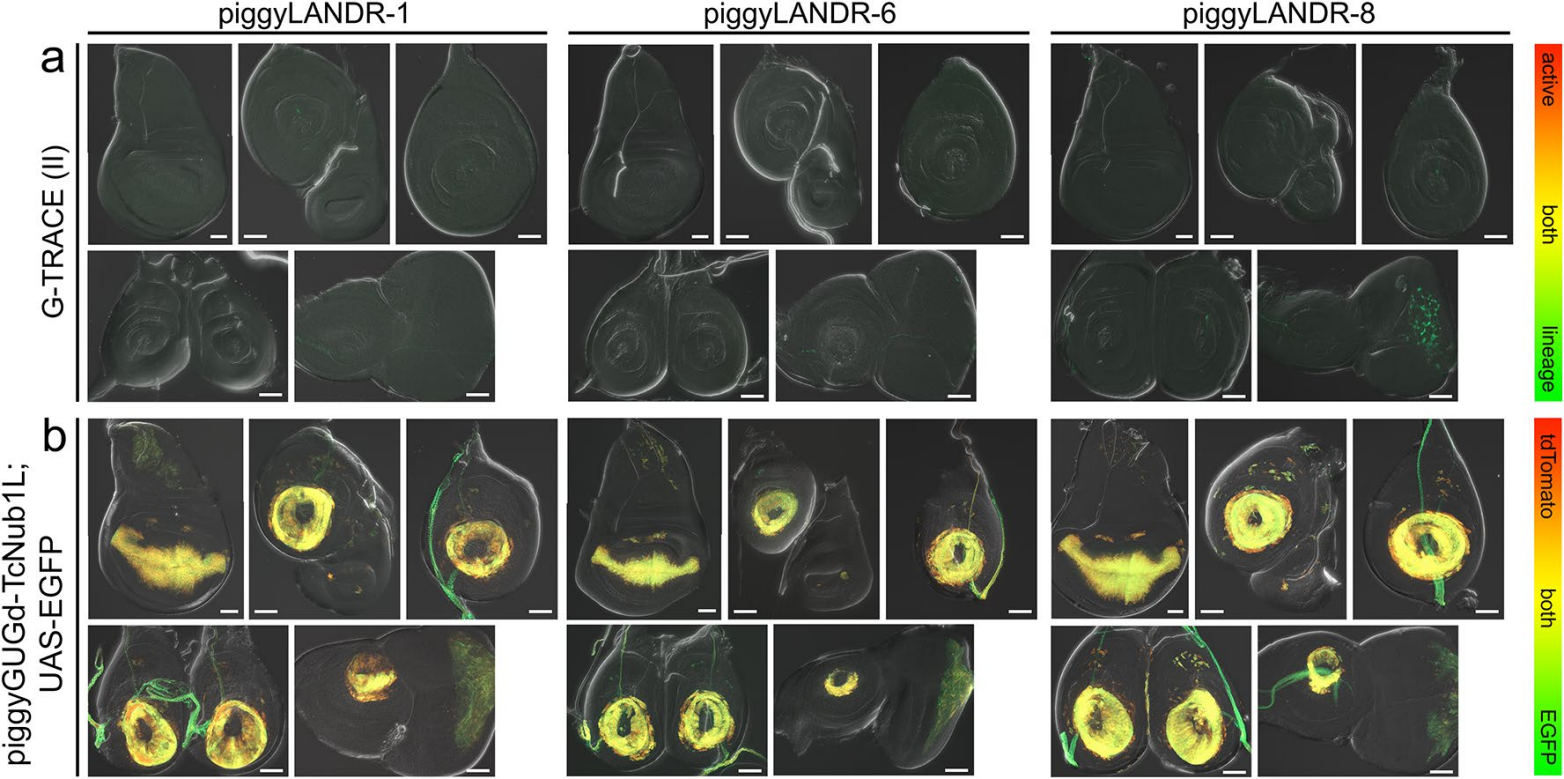
The lack of detectable enhancer traps and silencing effects at potential landing sites. (**a**) The lack of active and lineage trace expression in three piggyLANDR lines, except for some neurons in the eye of piggyLANDR-8. (**b**) EGFP and tdTomato expression of the three piggyLANDR lines when crossed to piggyGUGd-TcNub1L; UAS-EGFP. Overlapping expression of tdTomato and EGFP suggests no silencing effect on these loci in the developing appendages. Scale bars: 50 $\mu$m.

From this initial screen, three potentially suitable landing sites (piggyLANDR-1, 6, and 8) were selected based on minimal UAS-tdTomato and UAS-EGFP expression in the last larval stage (Figs. 6, 7, S1, S2, Table S3) for further analysis of silencing or activating position effects. First, these lines were tested for enhancer traps on DSCP-Gal4d at an earlier stage by crossing to the lineage-tracer G-TRACE^31^. With G-TRACE, enhancer activity drives expression of Flp recombinase, which removes a stop cassette between a ubiquitin promoter and EGFP coding sequence. This causes daughter cells to continue to express EGFP even after enhancer activity (and thus the expression of Gal4d) has ceased. No lines exhibited significant lineage tracing expression indicative of earlier activating position effects (Fig. 8a). The lineage tracing observed in the eye disc in piggyLANDR-8 (Fig. 8a) is suspected to result from the 3xP3 artificial enhancer acting on the DSCP, as observed with some other constructs (Fig. 2a–d, f–h). Next, we ensured that the UAS-tdTomato cassette was not silenced by crossing each line to a Gal4 driver that is active in all imaginal discs analyzed (piggyGUGd-TcNub1L, Fig. 2d) with UAS-EGFP as an internal control (Fig. 8b). UAS-tdTomato was activated similarly to UAS-EGFP indicating that there are negligible silencing position effects at these insertions (Fig. 8b). Since no activating or silencing position effects were detected, these three lines would make ideal candidates for future landing sites.

**Figure 9:**
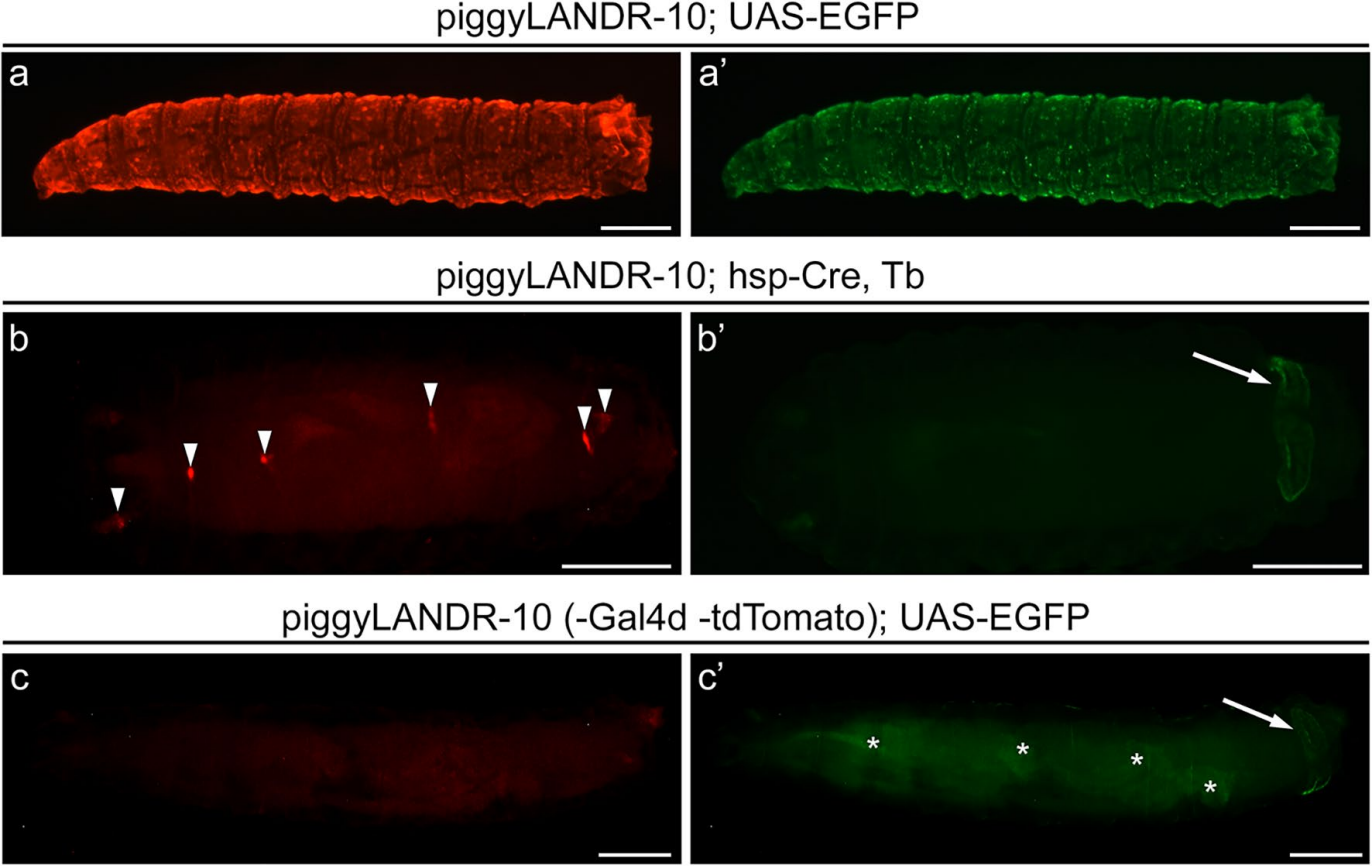
Removing the DSCP-Gal4d UAS-tdTomato cassette from piggyLANDR. (**a**, **a’**) Ubiquitous epidermal expression of Gal4d and tdTomato in piggyLANDR-10. (**b**, **b’**) Loss of UAS-tdTomato expression upon crossing with hsp-Cre, Tb, demonstrating successful removal of DSCP-Gal4d and/or UAS-tdTomato from piggyLANDR in all but a few small groups of cells (arrowheads, **b**). 3xP3-ECFP expression in the anal pads (arrow, **b**’) confirms that piggyLANDR is not simply absent. (**c**, **c’**) Confirmation of the removal of the DSCP-Gal4d UAS-tdTomato cassette from piggyLANDR-10 by UAS-EGFP. Lack of UAS-tdTomato (**c**) and UAS-EGFP (**c**’) expression indicates successful removal of both DSCP-Gal4d and UAS-tdTomato, while 3xP3-ECFP expression in the anal pads confirms the presence of piggyLANDR (arrow, **c**’). Some background expression of UAS-EGFP (independent of Gal4) is still visible (asterisks, **c**’). Scale bars: 0.5 mm.

The final step in establishing an attP landing site with piggyLANDR is the removal of the DSCP-Gal4d UAS-tdTomato cassette via Cre/loxP recombination. We performed this step in a line that exhibits a ubiquitous epidermal expression of Gal4d and tdTomato (piggyLANDR-10, Fig. 9a, a’) to simplify the downstream verification procedures (but the DSCP-Gal4d UAS-tdTomato cassette excision can be performed in any piggyLANDR lines with several additional steps). When piggyLANDR-10 was crossed to an hsp-Cre driver, tdTomato expression was eliminated in almost all epidermal cells in G0 individuals from this cross (Fig. 9b, b’). Five G0 individuals were then crossed separately to UAS-EGFP to ensure the removal of Gal4d. Four out of the five crosses resulted in no expression of tdTomato or EGFP, indicating the proper removal of both DSCP-Gal4d and UAS-tdTomato from piggyLANDR (Fig. 9c, c’).

Taken together, these results demonstrate that the piggyLANDR platform works properly to identify a proper landing site for PhiC31 integration in an insect genome. After identification of a landing site free of activating or silencing position effects (Fig. 8) and subsequent removal of DSCP-Gal4 and UAS-tdTomato (Fig. 9), a researcher would be left with an ideal 3xP3-ECFP marked PhiC31 landing site (Fig. 5c).

## Discussion

In this study, we created a suite of new enhancer-reporter vectors that can be used in diverse insect species. The availability of multiple reporter genes and transgenic markers will be useful when analyzing the activity of several enhancers simultaneously, as well as when needing to avoid a certain wavelength of background fluorescence observed in some insects. Furthermore, the dual compatibility of our vectors with *piggyBac* transgenesis and PhiC31 site-directed integration will be beneficial when studying enhancer activity in the cross-species setting, as one reporter construct can be used for two different transgenic methods based on the genetic tools available in certain species (for example, use PhiC31 site-directed integration in *Drosophila* and *piggyBac* transgenesis in *Tribolium*).

The choice of core promoters has been an issue when constructing a reliable enhancer-reporter assay system outside of *Drosophila*^7^. We have shown that our reporter constructs with the DSCP function properly in at least in three of the four major holometabolous orders: the Diptera (ref^19^; this study), the Coleoptera (ref^19^; this study), and the Lepidoptera^20^. In addition, piggyPhiGUE also worked in another dipteran insect, *Aedes aegypti*^21^. These outcomes suggest that the reporter constructs presented in this study may function well in various insect species in Holometabola, and perhaps even in hemimetabolous species.

Avoiding position effects is another significant point when evaluating enhancer activity through a reporter assay. Extensive work has been done to precisely characterize the silencing and activating position effects on PhiC31 attP landing sites in the *Drosophila* genome^26^. We developed piggyLANDR, which allows surveying for PhiC31 landing sites free from position effects based on expression of several reporter genes. As a proof-of-principle, we demonstrated three primary functions of piggyLANDR in *Drosophila*: (i) detect enhancer traps using the DSCP-Gal4d/UAS-tdTomato (Figs. 6–8a), (ii) assess silencing via activation of the UAS-tdTomato with a separate Gal4 driver (Fig. 8b), and (iii) neutralize the reporter function upon Cre-loxP recombination to leave a 3xP3-ECFP marked attP landing site (Fig. 9). Although the piggyLANDR platform requires established Cre-loxP and Gal4/UAS systems (thus currently is applicable only to a handful of insects in which these systems have been implemented, such as *Tribolium*^16,32^, *Bombyx mori*^33,34^, and others^35–39^), once established, this workflow will allow for preliminary analyses to eliminate unsuitable landing sites prior to enhancer-reporter studies.

The need for such a system to avoid position effects at landing sites is evident in the diverse enhancer-trap driven expression patterns obtained from our independent *Drosophila* piggyLANDR lines (Figs. 6–7, S1–S2, Table S3). Further, the utilization of more than one core promoter by our system allowed the detection of differential silencing and activating position effects on different core promoters at the same site. We observed cases of activating effects on the DSCP with simultaneous silencing effects on the Tc-bhsp68 (in the epidermis and salivary glands of lines 3b and 7, respectively; Fig. 6, Table S3 yellow cells), as well as instances of the inverse case, with silencing effects on the DSCP but activating effects on the Tc-bhsp68 (in the larval testis of lines 8 and 10 and the imaginal discs of line 10; Figs. 6–7, Table S3 orange cells). The frequency of both the simple and complex position effects observed with this system highlights the necessity of carefully surveying genomes for suitable landing sites for site-specific integration strategies.

As with the previously published mCherry reporter, piggyGUM^19^, our new EGFP reporter piggyGUE was fully functional in both *Drosophila* and *Tribolium* (Figs. 2, 3). While our fluorescent gene reporters behaved similarly both in *Tribolium* and *Drosophila* (Figs. 2, 3), complications arose with our Gal4-UAS constructs in *Tribolium* (Fig. 4). Despite using the Gal4d deletion variant and *Tc-hsp68* core promoter in the UAS-tdTomato cassette, previously shown to be required for function in *Tribolium*^16^, in our hands, these constructs were unable to drive tissue-specific expression consistent with the direct reporters (Figs. 3, 4). There are several potential explanations for this discrepancy. Our Gal4-UAS constructs may contain an accidentally created artificial CRE, which could have altered Gal4 expression. Alternatively, the *Tc-hsp68* core promoter at the UAS might be activated non-specifically in certain tissues but silenced in others. Regardless, further modifications to the Gal4-UAS system will be required to establish a reliable tissue-specific binary expression system in *Tribolium*. It will also be interesting to try other binary expression systems in *Tribolium*, such as QF-QUAS^40^, LexA-LexAop^41^, or TALE-based systems^42^.

Here, we presented new tools for the study of enhancers in non-traditional model insects. Our constructs will facilitate the study of *cis* properties of GRNs outside of *Drosophila*, providing novel insights into development, evolution, and disease. These constructs also provide a platform for the expression of exogenous genes outside of *Drosophila* for additional purposes such as pest management and disease vector control*.*

## Materials and methods

### Vector construction: *piggyBac* vectors

To create **piggyGUE-DEST**, an [attR1-ccdB-chl^*r*^-attR2 DSCP-EGFP-Dm-hsp70polyA] cassette flanked by AscI and FseI enzyme sites was de novo synthesized and cloned in to pBac[3xP3-dsRed]af^43^ (Genscript, Piscataway, NJ). Note that, pBac[3xP3-dsRed]af, and thus, piggyGUE and piggyPhiGUE, contain a shortened but functional version of the left *piggyBac* arm^44^. **piggyGUG-DEST** was also constructed via de novo synthesis, with an [attR1-ccdB-chl^*r*^-attR2 DSCP-Gal4-Dm-hsp70polyA] cassette flanked by AscI and FseI enzyme sites cloned into pBac[3xP3-EGFP]af (Genscript, Piscataway, NJ). For testing purposes, piggyGUGd-TcNub1L was created prior to the destination version. To create piggyGUGd-TcNub1L, primers with 15 bp of reverse complementary 5’ overlap were used to amplify outwards from the coding regions of the DBD and AD of Gal4 in piggyGUG-TcNub1L and fused via seamless cloning (GeneArt™ Seamless Cloning and Assembly Kit, Invitrogen, cat #A13288). To generate **piggyGUGd-DEST**, a fragment within Gal4d overlapping an XhoI site in the DBD and an RsrII site in the AD was PCR amplified from piggyGUGd-TcNub1L and cloned into the corresponding sites in piggyGUG-DEST.

### Vector construction: *piggyBac* + PhiC31 vectors

**piggyPhiGUE-DEST** was created using primers with reverse complementary overlap at their 3’ ends comprising the sequence of a PhiC31 attB site flanked by NdeI sites. These overlapping primers were extended via PCR, then digested and cloned into a single NdeI site in piggyGUE-DEST. To construct **piggyPhiGUGd-DEST**, primers with reverse complementary overlap at their 3’ ends which comprise the sequence of a PhiC31 attB site flanked by an MCS (AvrII, NheI, PacI) and NdeI sites were extended via PCR, then digested and cloned into a single NdeI site in piggyGUGd-DEST. For **piggyPhiGUGdTomO-DEST,** a [loxP-12xUAS-Tc-bhsp68-tdTomato-SV40-loxP] cassette flanked by NheI sites was de novo synthesized (Genscript, Piscataway, NJ) and cloned into a single NheI site in the MCS adjacent to the PhiC31 attB site outside the *piggyBac* arms of piggyPhiGUGd-DEST. In the case of **piggyPhiGUGdTomI-DEST**, the same cassette was flanked by AscI sites and cloned into a single AscI site within the *piggyBac* arms of piggyPhiGUGd-DEST (Genscript, Piscataway, NJ). To create **piggyPhiGUGdTomIB-DEST**, primers containing an ApaI site, AscI site, and an additional unique sequence, were designed to amplify the ECFP-SV40 sequence from pBac[6xP3-Tc-hsp-ECFP-SV40] (Addgene Plasmid #86,447) and cloned into a single ApaI site and one of the two AscI sites in piggyPhiGUGdTomI-DEST. To generate **piggyLANDR**, an [attL1-PhiC31 attP-loxP-attL2 cassette] was de novo synthesized (Genscript, Piscataway, NJ) and introduced upstream of the DSCP in piggyPhiGUGdTomIB-DEST via GATEWAY cloning. The absence of the enhancer-independent reporter activity was confirmed in the *Drosophila* larval tissues using a negative control construct (piggyPhiGUGd-NC, in which the GATEWAY cassette was removed from piggyPhiGUGd-DEST without inserting any enhancer sequence), although we saw some vector-driven reporter gene expression during embryogenesis^46^. A similar negative control construct should be used to test the possibility of vector-driven reporter gene expression when using our reporter constructs in a new species.

### Enhancer GATEWAY cloning

Except for piggyGUGd-TcNub1L, the TcNub1L enhancer was introduced into all constructs via GATEWAY cloning as previously described (Lai et al., 2018).

#### Transgenics

For *Drosophila* transgenesis, PhiC31-compatible constructs were transformed into the attP2 site (68A4) of strain 8622, while *piggyBac*-only-compatible constructs were randomly integrated into strain w^1118^, using 3xP3-ECFP, 3xP3-EGFP, or 3xP3-dsRed as a transgenic marker (Best Gene Inc, USA). For *Tribolium* transgenesis, random *piggyBac*-mediated integration was used to introduce constructs into strain *vermillion*^*white*^ using 3xP3-EGFP or 3xP3-dsRed as a transgenic marker (Trigenes, Goettingen, DE).

#### Drosophila stocks

Three *Drosophila* strains used in this study were obtained from the Bloomington *Drosophila* Stock Center: **G-TRACE(II)** (RRID:BDSC_28280): P{UAS-RedStinger}4, P{UAS-Flp}JD1, P{Ubi-p63E(FRT.STOP)Stinger} 9F6/CyO, **20xUAS-6xGFP** (RRID:BDSC_52262): P{20xUAS-6xGFP}attP2, **hsp-Flp/hsp-Cre** (RRID:BDSC_1501): P{hsp-Flp}, MKRS/ P{hsp-Cre}, Tb.

#### *Tribolium* cultures

Beetle cultures were reared on whole-wheat flour (+ 5% yeast) at 30 °C in a temperature- and humidity-controlled incubator. For heat shock, piggyGUG-TcNub1L pupae were placed in an open plastic dish in a hybridization oven set to 48C for 30 min. *pu11* is a *Tc-nub* enhancer trap line that expresses EYFP in the wing tissue and was used as a comparator for the function of TcNub1L constructs in *Tribolium*^14,19,45^.

### Tissue preparation

*Drosophila* and *Tribolium* imaginal discs were dissected from last-instar larvae in 1X Phosphate Buffered Saline (PBS) (MP Biomedicals LLC, cat #2,810,305) and fixed in 1X PBS, 4% formaldehyde (Polysciences Inc., cat #04,018–1) at room temperature for 20 or 30 min, respectively. Discs were washed in 1X PBS, 0.1% Triton™ X-100 (Sigma-Aldrich, cat #T8787-50ML) and mounted on glass slides with ProLong™ Gold antifade reagent (Life Technologies, cat #P36934) for documentation.

### Image processing and documentation

Mounted discs were imaged with a Zeiss AxioCam 503 color on a Zeiss Axio Imager.M2 with an ApoTome.2. Raw apotome z-stack images (4 phased images per slice) were processed for phase correction and local bleaching prior to orthogonal projection via the Maximum Intensity method in Zen. Whole larvae and pupae were imaged using a Zeiss AxioCam MRc5 on a Zeiss SteREO Discovery.V12.

### Supplementary Information

Supplementary Information.

## Data Availability

The sequences of the reporter vectors established during the current study are available in the Genbank database [accession numbers: piggyGUE_DEST (PP373646), piggyGUG_DEST (PP373647), piggyGUGd_DEST (PP373648), piggyGUM_DEST (PP373649), piggyPhiGUE_DEST (PP373650), piggyPhiGUGd_DEST (PP373651), piggyPhiGUGdTomI_DEST (PP373652), piggyPhiGUGdTomIB_DEST (PP373653), piggyPhiGUGdTomO_DEST (PP373654), piggyLANDR (PP373655)]. These vectors can be obtained from The *Drosophila* Genomics Resource Center (https://dgrc.bio.indiana.edu).
