# Supplementary material for: A new suite of reporter vectors and a novel landing site survey system to study cis-regulatory elements in diverse insect species
Source: Sci Rep. 2024 May 2;14:10078. doi: 10.1038/s41598-024-60432-9 (PMC11066043; doi:10.1038/s41598-024-60432-9)
Supplement: Supplementary file 1 — Supplementary Information. [file 41598_2024_60432_MOESM1_ESM.pdf]

A new suite of reporter vectors and a novel landing site survey system to study *cis*-regulatory elements in diverse insect species

Kevin D. Deem[1, #], Marc S. Halfon[2], and Yoshinori Tomoyasu[1,*]
[1]Department of Biology, Miami University, Oxford, OH 45056, USA
[2]Department of Biochemistry, University at Buffalo-State University of New York, Buffalo, NY 14203, USA
* Corresponding author. Email address: tomoyay@miamioh.edu
# Present address: Department of Biology, University of Rochester, Rochester, NY 14627, USA

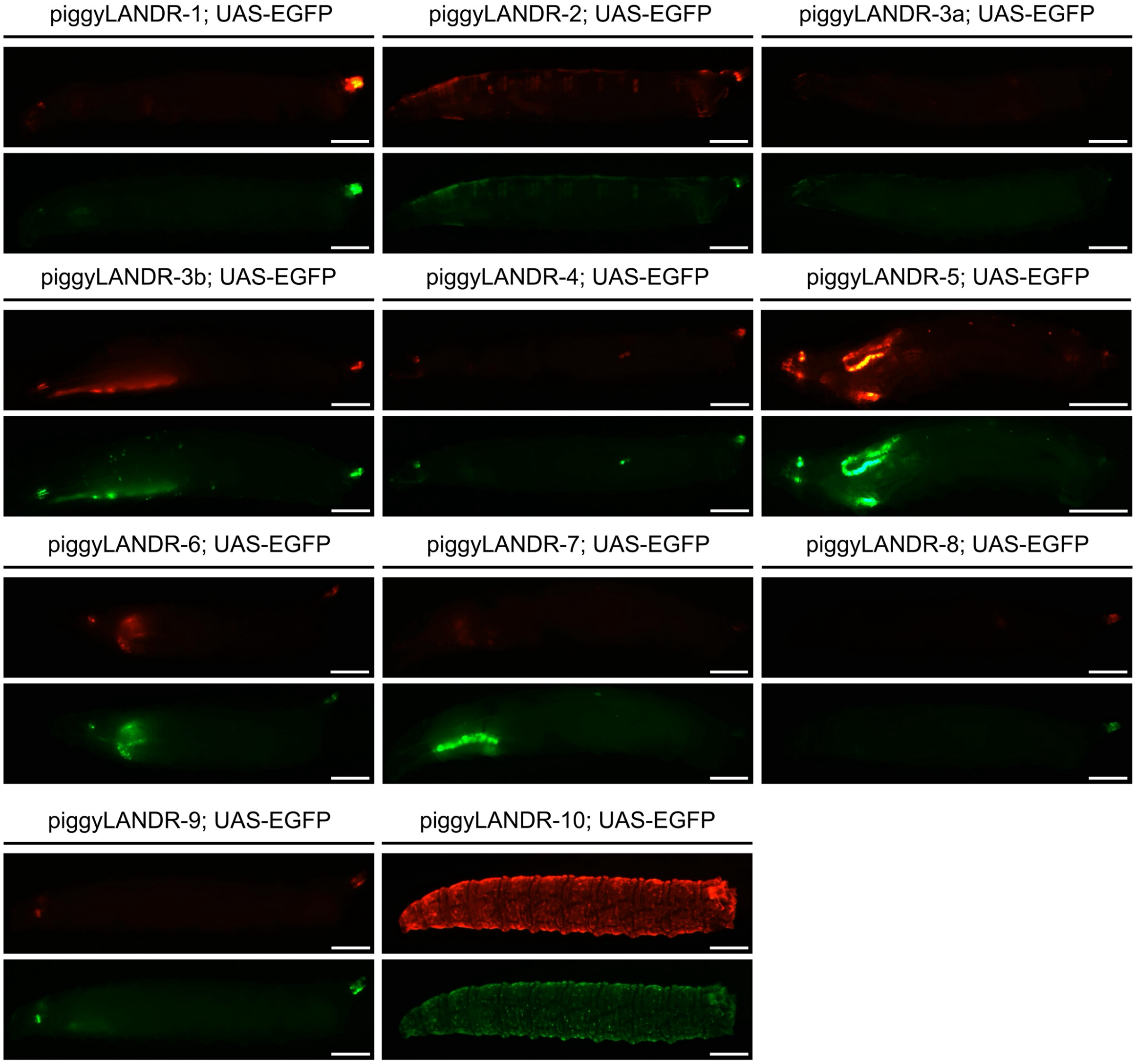

Fig. S1 Enhancer traps throughout the body for all 11 piggyLANDR lines
External view of enhancer trap expression for all 11 piggyLANDR lines crossed with UAS-EGFP. Six lines (piggyLANDR-2, 3b, 5, 6, 7, and 10) exhibited prominent enhancer trap expression visible from the exterior. Scale bars: 0.5mm

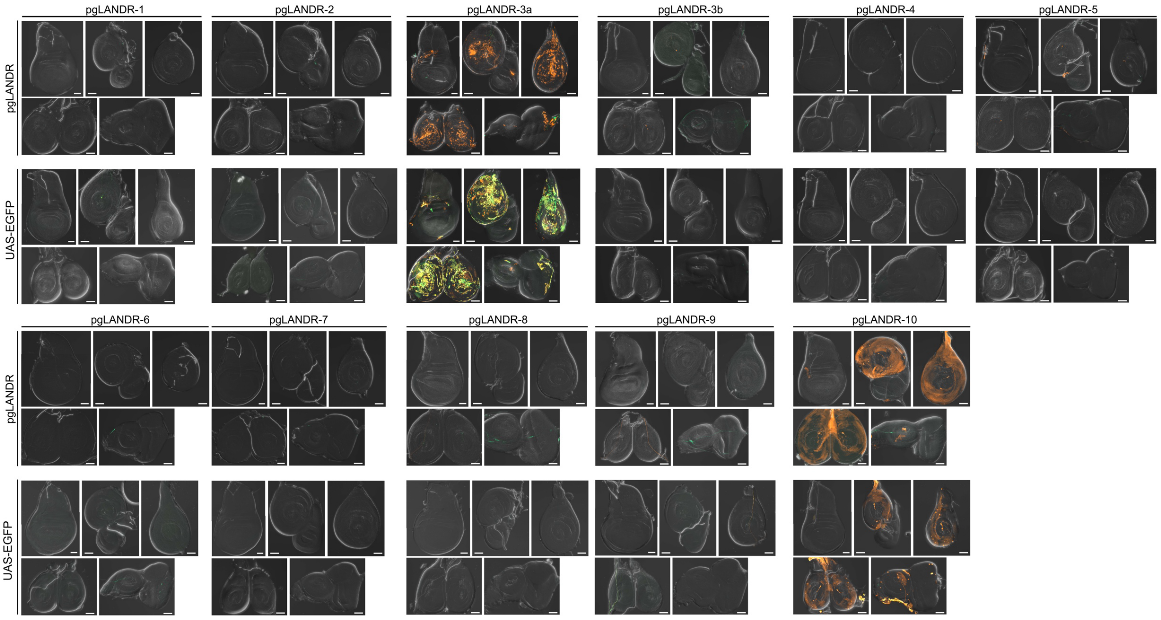

Fig. S2 piggyLANDR expression in larval imaginal discs for all 11 lines
Enhancer trap expression within the third instar larval imaginal discs of all 11 piggyLANDR lines crossed with UAS-EGFP. Two lines (piggyLANDR-3a and -10) exhibited significant enhancer trap activity in the imaginal discs, making these two insertion sites unsuitable for use as PhiC31 landing sites. Scale bars: 50μm

Vector	Transgenesis	GATEWAY compatible	Core Promoter(s)	Reporter(s)	Marker
piggyGUM	*piggyBac* only	Y	DSCP	mCherry	3xP3-EGFP
piggyGUE	*piggyBac* only	Y	DSCP	EGFP	3xP3-dsRed
piggyGUG	*piggyBac* only	Y	DSCP	Full-length Gal4	3xP3-EGFP
piggyGUGd	*piggyBac* only	Y	DSCP	Gal4-delta	3xP3-EGFP
piggyPhiGUE	*piggyBac* and PhiC31	Y	DSCP	EGFP	3xP3-dsRed
piggyPhiGUGd	*piggyBac* and PhiC31	Y	DSCP	Gal4-delta	3xP3-EGFP
piggyPhiGUGdTomI	*piggyBac* and PhiC31	Y	DSCP/ Tc-bhsp68	Gal4-delta/ tdTomato	3xP3-EGFP
piggyPhiGUGdTomO	PhiC31 only for UAS	Y	DSCP/ Tc-bhsp68	Gal4-delta/ tdTomato	3xP3-EGFP
piggyPhiGUGdTomIB	*piggyBac* and PhiC31	Y	DSCP/ Tc-bhsp68	Gal4-delta/ tdTomato	3xP3-ECFP
piggyLANDR	*piggyBac* only	N	DSCP/ Tc-bhsp68	Gal4-delta/ tdTomato	3xP3-ECFP

Table S1: Vector names and components

A table of all vector names and core components in the order they are referred to in the name (except for piggyLANDR): ***piggy**Bac* arms (piggy), **Phi**C31 attB site (Phi), **G**ateway cloning cassette (G), **U**niversal *Drosophila* Synthetic Core Promoter (U), Reporter protein (**M**: mCherry, **E**: EGFP, **G**: Gal4, **Gd**: Gal4-delta), UAS-tdTomato **I**nside (TomI) or **O**utside (TomO) of the *piggyBac* arms, the **B**lue eye marker of 3xP3-ECFP (B).

Primer name	5'-3' sequence	Construct
Gal4dF1smls	TGTATCGGCCAATTTTAATCAAAGTGGG	piggyGUGd-TcNub1L
Gal4dR1smls	AAATTGGCCGATACAGTCAACTGTCTTTG	piggyGUGd-TcNub1L
Gal4digestF	ATCGCCTCGAGAAGACCTTGAC	piggyGUGd-empty
Gal4R3	GAGGTTCGGACCGTTGCTACTG	piggyGUGd-empty
NdeI_attB_F	ATCGCATATGCTCGAAGCCGCGGTGCGGGTGCCAGGGCGTGC CCTTGGGCTCCCC	piggyPhiGUE-empty
NdeI_attB_R	CGATCATATGGATGGGTGAGGTGGAGTACGCGCCCGGGGAGC CCAAGGGCACGCC	piggyPhiGUE-empty
pgPhiGUGdeltaMCSF	ATCGCATATGCCTGAGGGACTGGTGACCCTCGAAGCCGCGGT GCGGGT	piggyPhiGUGd-empty
pgPhiGUGdeltaMCSR	ATCGCATATGTTAATTAACGATGCTAGCAGCTCCTAGGGATGG GTGAGGTGGAGTACG	piggyPhiGUGd-empty
ECFP-SV40digF1	ATGCGGGCCCGGTACCGGTCGCCACCATGG	piggyPhiGUGdTomIB-empty
KDDEEM_dig_R1	CTAGGCGCGCCCATCTCTTCGTCATCCTTAGCTTTAAGATACAT TGATG	piggyPhiGUGdTomIB-empty

Table S2: List of primers used in vector construction
Primers used in this study, along with 5'-3' sequence and the vector they were used to construct.

	Reporter expression in third instar larvae due to position effects												
piggy LANDR Line	CNS	Salivary Gland	Muscle	Epidermis	Testis	Posterior Spiracle	Unidentified Internal	Eye/antennal disc	T1 leg disc	T2 leg disc	T3 leg disc	Wing disc	Haltere disc
1	N / N	N / N	N / N	N / N	N / N	**Y / Y**	N / N	N / N	N / N	N / N	N / N	N / N	N / N
2	N / N	N / N	**Y / Y**	N / N	N / N	**Y / Y**	N / N	N / N	N / N	N / N	N / N	N / N	N / N
3a	N / N	N / N	N / N	N / N	N / N	N / N	N / N	**Y / Y**	**Y / Y**	**Y / Y**	**Y / Y**	**Y / Y**	**Y / Y**
3b	N / N	**Y / Y**	N / N	**Y / N**	N / N	**Y / Y**	N / N	N / N	N / N	N / N	N / N	N / N	N / N
4	N / N	N / N	N / N	N / N	N / N	**Y / Y**	N / N	N / N	N / N	N / N	N / N	N / N	N / N
5	**Y / Y**	**Y / Y**	N / N	N / N	N / N	**Y / Y**	**Y / Y**	N / N	N / N	N / N	N / N	N / N	N / N
6	N / N	N / N	N / N	N / N	N / N	**Y / Y**	**Y / Y**	N / N	N / N	N / N	N / N	N / N	N / N
7	N / N	**Y / N**	N / N	N / N	N / N	N / N	N / N	N / N	N / N	N / N	N / N	N / N	N / N
8	N / N	N / N	N / N	N / N	**N / Y**	**Y / Y**	N / N	N / N	N / N	N / N	N / N	N / N	N / N
9l	N / N	N / N	N / N	N / N	N / N	**Y / Y**	N / N	N / N	N / N	N / N	N / N	N / N	N / N
10	N / N	N / N	N / N	**Y / Y**	**N / Y**	N / N	N / N	**N / Y**	**N / Y**	**N / Y**	**N / Y**	**N / Y**	**N / Y**

Table S3: Position effects on individual piggyLANDR core promoters

Reporter expression in third instar larval tissues caused by local activating or silencing effects on the two core promoters, DSCP or Tc-bhsp68, in piggyLANDR insertion lines. Cells are shaded green when DSCP-Gal4d was activated with no silencing of Tc-bhsp68-UAS-tdTomato, yellow when there was an activating effect on DSCP-Gal4d but a silencing effect on Tc-bhsp68-UAS-tdTomato, and orange in cases where DSCP-Gal4d was silenced but Tc-bhsp68-UAS-tdTomato was activated. The minimal overall enhancer trap activity of piggyLANDR-1 and -4 makes these insertion sites good candidates for ideal PhiC31 landing sites free of position effects.
